# Visual acuity and foveal thickness after vitrectomy for macular edema associated with branch retinal vein occlusion: a case series

**DOI:** 10.1186/1471-2415-10-11

**Published:** 2010-04-29

**Authors:** Hidetaka Noma, Hideharu Funatsu, Tatsuya Mimura, Shuichiro Eguchi, Katsunori Shimada

**Affiliations:** 1 Department of Ophthalmology, Yachiyo Medical Center, Tokyo Women's Medical University, Chiba, Japan; 2 Department of Ophthalmology, University of Tokyo Graduate School of Medicine, Tokyo, Japan; 3 Department of Ophthalmology, Eguchi Eye Hospital, Hakodate, Japan; 4 Department of Hygiene and Public Health II, Tokyo Women's Medical University, Tokyo, Japan

## Abstract

**Background:**

The mechanism by which vitrectomy improves macular edema in patients with branch retinal vein occlusion remains unclear, although intraocular levels of vascular endothelial growth factor have been suggested to influence the visual prognosis and macular edema.

**Methods:**

A series of 54 consecutive patients (54 eyes) with branch retinal vein occlusion was studied prospectively. All patients underwent pars plana vitrectomy for treatment of macular edema. Best corrected visual acuity and retinal thickness (examined by optical coherence tomography) were assessed before and after surgery. The level of vascular endothelial growth factor in vitreous fluid harvested at operation was determined. Patients were followed for at least 6 months postoperatively.

**Results:**

Both the visual acuity and the retinal thickness showed significant improvement at 6 months postoperatively (P = 0.0002 and P < 0.0001, respectively). The vitreous level of vascular endothelial growth factor was significantly higher in patients who showed less improvement of visual acuity compared with those who had a better visual prognosis (p = 0.0135). In contrast, a high vitreous level of vascular endothelial growth factor was associated with greater improvement of macular edema (p = 0.0064).

**Conclusions:**

These results suggest that the vitreous level of vascular endothelial growth factor might influence the visual prognosis and the response of macular edema to vitrectomy in patients with branch retinal vein occlusion.

## Background

Branch retinal vein occlusion (BRVO) is a common retinal vascular disease that often causes macular edema, which is the main reason for visual impairment in these patients[1,2]. We have been investigating various factors that might be involved in the pathogenesis of macular edema associated with BRVO. We recently reported that the vitreous level of vascular endothelial growth factor (VEGF) is elevated in BRVO patients with macular edema, and that vitreous VEGF levels are correlated with the non-perfused area of the retina and with the severity of macular edema[3,4]. VEGF directly increases vascular permeability[5], and VEGF expression by retinal glial cells is upregulated due to hypoxia[6]. Accordingly, VEGF may contribute to the occurrence of macular edema in patients with BRVO, and a decrease of VEGF production may be related to the improvement of macular edema in BRVO patients who undergo pars plana vitrectomy (PPV).

We recently reported that higher VEGF levels in the vitreous fluid were associated with more marked improvement of macular edema after PPV in patients with BRVO[7]. However, the relations between VEGF, the visual prognosis, and the improvement of macular edema after PPV need to be investigated in more detail. In the present study, we evaluated the outcome of PPV for macular edema in patients with BRVO and examined the relations between VEGF in vitreous fluid, the final best corrected visual acuity, and the response of macular edema to PPV.

## Methods

### Subjects

The subjects all underwent PPV at Tokyo Women's Medical University Hospital or Eguchi Eye Hospital and undiluted vitreous fluid samples were harvested at the start of surgery. Written informed consent was obtained from each subject after an explanation was given about the purpose and potential adverse effects of the procedure. This study was performed in accordance with the Helsinki Declaration of 1975 (1983 revision). The institutional review boards of Tokyo Women's Medical University and Eguchi Eye Hospital both approved the protocol for collection and testing of vitreous fluid. Consecutive patients presenting with BRVO between June 2005 and November 2008 were screened according to the following criteria and 56 patients were enrolled. The indications for pars plana vitrectomy were: (1) clinically detectable diffuse macular edema or cystoid macular edema persisting for more than 3 months, (2) best-corrected visual acuity worse than 20/40, and (3) macular edema that persisted after retinal photocoagulation. Significant macular edema was defined as retinal thickening that covered at least one optic disc area and involved the fovea[8]. The exclusion criteria for this study were (1) ocular surgery within the previous 6 months, (2) diabetic retinopathy, (3) iris rubeosis, (4) a history of ocular inflammation or vitreoretinal disease, and (5) the reason being previous ocular or vitreous injection of anti-VEGF agent. Patients with iris rubeosis were excluded because high cytokine levels have been reported in this disease[9] and because its pathology may differ from that of macular edema. Twenty-nine of the 54 patients underwent both PPV and cataract surgery. During the 6-month postoperative follow-up period after surgery, cataract extraction was not performed in the other 25 patients who did not have cataracts at baseline and additional scatter laser photocoagulation was also not done.

### Ocular investigations

Best-corrected visual acuity was measured before and after PPV in decimal units and the data were converted to the logarithm of the minimum angle of resolution (log MAR) scale. Biomicroscopic examination was performed with a fundus contact lens. Fundus findings were confirmed preoperatively by standardized fundus color photography and fluorescein angiography, which was performed with a Topcon TRC-50EX fundus camera, an image-net system (Tokyo Optical Co. Ltd., Japan), and a preset lens with a slit-lamp.

A masked grader independently assessed ischemic retinal vascular occlusion on fluorescein angiograms. The ischemic region of the retina was measured with the public domain Scion Image program, as reported previously[3,4,10]. In brief, the disc area was outlined on a digital fundus photograph using a cursor and then was measured. Next, the non-perfused area was outlined and sites of retinal photocoagulation were excluded when calculating the non-perfused area. Finally, the non-perfused area was divided by the disc area as an index of retinal ischemia.

Optical coherence tomography (OCT) (Zeiss-Humphrey Ophthalmic Systems, Dublin, California, USA) was performed in each subject within 1 week before surgery. The fundi were scanned with the beam focused on horizontal and vertical planes that crossed the central fovea, which was located on the fundus photograph and by the patient fixing on the central landmark during OCT. (All subjects were able to maintain fixation.) Cross-sectional images were collected by a single experienced examiner, who continued each study until reproducible scans were obtained. The thickness of the central fovea was defined as the distance between the inner limiting membrane and the retinal pigment epithelium (including any serous retinal detachment), while the thickness of the neurosensory retina was defined as the distance between the inner and outer neurosensory retinal surfaces[11]. The severity of macular edema was determined from the measured retinal thickness and all measurements were obtained automatically by computer analysis.

### PPV and measurement of VEGF

Under local anaesthesia, all patients underwent standard three-port PPV. All epiretinal material, the residual cortex, and the posterior hyaloid were removed from the retina around the macula as completely as possible with the assistance of triamcinolone acetonide, which was injected into the eyeball at the minimum amount required and then was removed as thoroughly as possible at the end of surgery, because it can influence cystoid macular edema. Scatter laser photocoagulation was also applied intraoperatively to the ischemic region of the retina in 24 eyes (mean: 237 shots; range: 57 to 350 shots). Peeling of the internal limiting membrane was not performed. All patients were followed for at least 6 months postoperatively.

Samples of undiluted vitreous fluid (300-500 μl) were collected into sterile tubes at the time of surgery and were rapidly frozen at - 80°C for storage until VEGF was measured by an enzyme-linked immunosorbent assay using a kit for human VEGF (R&D Systems, Minneapolis, MN, USA)[3,4,10]. This assay detected two of the four isoforms of VEGF (VEGF_121 _and VEGF_165_). The minimum detectable concentration of VEGF was 15.6 pg/ml (intra-assay coefficient of variation: 5.4%; inter-assay coefficient: 6.8%), so the VEGF level in vitreous fluid samples was within the detection range of the assay.

### Statistical analysis

Analyses were performed with SAS System 9.1 software (SAS Institute Inc., Cary, North Carolina, USA). Data are presented as frequencies or as the mean ± SD. The paired *t*-test was used to compare the retinal thickness and the best-corrected visual acuity between the preoperative values and those obtained 6 months after surgery. To investigate the relation between each of the factors assessed and macular edema, Spearman's rank-order correlation coefficients were calculated. To identify factors with an influence on the visual prognosis, multiple linear regression analysis was performed, with the effects of profile, linearity, interaction, and collinearity on the multiple linear models being examined by regression diagnostic analysis. In all analysis, a two-tailed P value of less than 0.05 was taken to indicate statistical significance.

## Results

### Clinical profile

Of the 56 patients who were enrolled, 2 patients were lost to follow-up because of transfer to another hospital for personal reasons. The remaining 54 patients included 21 men and 33 women. Their mean age was 64.6 ± 10.0 years (range: 40-87 years), and the mean duration of BRVO before surgery was 4.5 ± 2.1 months (range: 3-10 months). Preoperative photocoagulation was done in 26 eyes (mean: 320 shots; range: 46 to 932). Retinal photocoagulation was done within 2.3 ± 1.5 months (range: 1 to 5 months) before surgery. None of the subjects received treatment with anti-VEGF agents or triamcinolone acetonide prior to surgery.

### Visual acuity and foveal thickness after PPV

At the initial examination, the mean best-corrected visual acuity was log MAR 0.84 ± 0.40, and it improved significantly to log MAR 0.54 ± 0.39 by 6 months after PPV (P = 0.0002). Four patients (7%) showed deterioration of their visual acuity, including two who had persistent macular edema after surgery and two with macular atrophy. The initial mean foveal thickness was 554. ± 182. μm and it decreased significantly to 308. ± 130. μm by 6 months after PPV (p < 0.0001). During PPV, a peripheral retinal tear occurred in three eyes, but these tears were successfully managed by endolaser photocoagulation and sulfur hexafluoride gas tamponade. Postoperatively, neovascular glaucoma was not detected in any of the patients after follow up for six months.

### Foveal thickness and the vitreous level of VEGF

**Figure 1:**
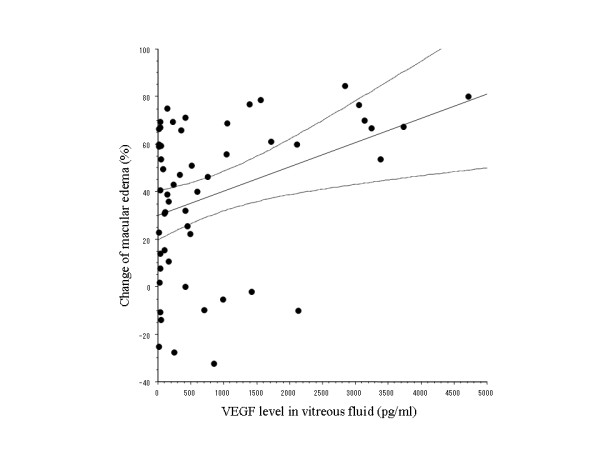
**Relation between vascular endothelial growth factor (VEGF) ad macular edema**. There is a significant positive correlation between the vitreous level of VEGF and the percent change of macular edema (r = 0.3666, p = 0.0064).

The percent change of macular edema (%ΔME) was calculated as follows:

where ME_pr _and ME_po _correspond to the foveal thickness before vitrectomy and 6 months after surgery, respectively. There was a significant positive correlation between the vitreous level of VEGF and %ΔME, with VEGF levels being significantly higher in patients who showed more marked improvement of macular edema after PPV (r = 0.3666, p = 0.0064, Figure 1).

### Visual acuity and the vitreous level of VEGF

**Figure 2:**
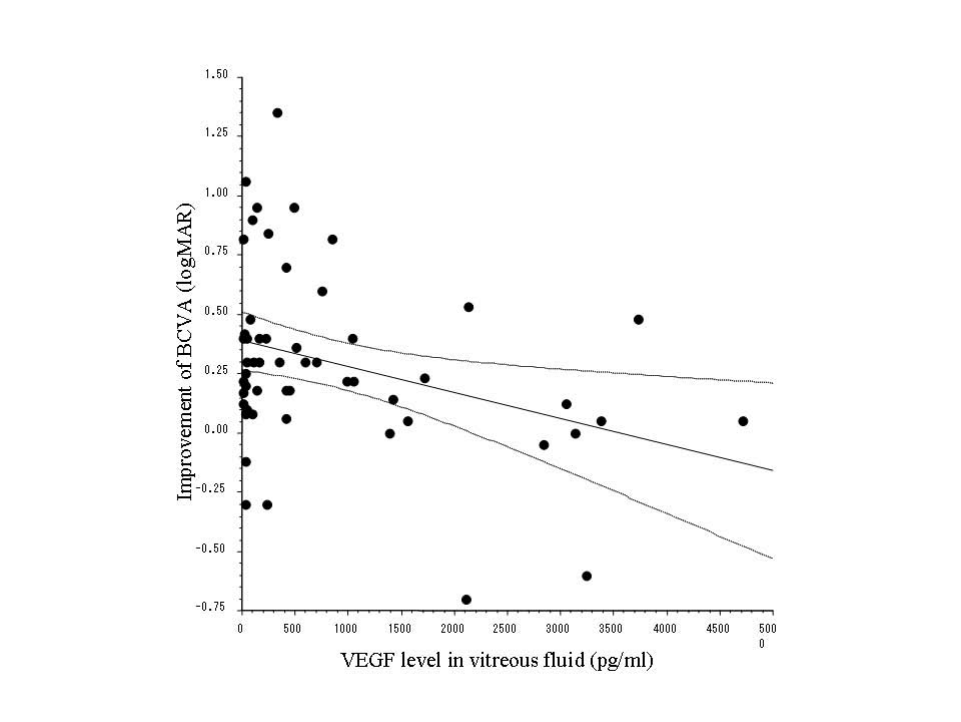
**Relation between vascular endothelial growth factor (VEGF) and visual acuity**. There is a significant negative correlation between the vitreous level of VEGF and the improvement of best-corrected visual acuity (BCVA) (r = -0.3342, p = 0.0135).

The improvement of best-corrected visual acuity was calculated by subtracting the postoperative visual acuity from the preoperative acuity. There was a significant negative correlation between the vitreous level of VEGF and the improvement of visual acuity, with a significantly lower VEGF level in patients who showed greater improvement of their visual acuity after PPV (r = - 0.3342, p = 0.0135, Figure 2).

### Relations between visual acuity, foveal thickness, and clinical factors

The following factors were investigated for an influence on best-corrected visual acuity and the foveal thickness: gender, age, hypertension, duration of BRVO, retinal photocoagulation, and the vitreous level of VEGF. As a result, the vitreous level of VEGF and performance of retinal photocoagulation were found to be significantly correlated with the improvement of visual acuity and with the decrease of foveal thickness (p = 0.0282 and p = 0.0026, p = 0.0286 and p = 0.0419, respectively) (Table 1).

**Table 1:** Results of multivariate analysis of factors influencing the improvement of best-corrected visual acuity (BCVA) and the decrease of foveal thickness.

	Improvement of BCVA (log MAR)		Decrease of foveal thickness	
**Variable**	**Standard partial** **regression coefficient**	**P *value***	**Standard partial** **regression coefficient**	**P *value***

Age (years)	0.166	0.1972	0.004	0.9749
Gender	0.114	0.3722	0.226	0.0803
Hypertension	0.098	0.4577	0.081	0.5345
Duration of BRVO (months)	-0.195	0.1497	-0.108	0.4190
Cataract surgery	0.039	0.7600	-0.068	0.5944
Retinal photocoagulation	0.307	0.0253	-0.273	0.0449
Vitreous VEGF level (pg/ml)	0.296	0.0270	0.422	0.0020

log MAR = logarithm of the minimum angle of resolution; BRVO = branch retinal vein occlusion; VEGF = vascular endothelial growth factor

Analysis of the 26 patients who had laser surgery prior to vitrectomy showed that the vitreous level of vascular endothelial growth factor was not associated with improvement of macular edema or improvement of visual acuity (r = 0.3826, p = 0.0572 and r = - 0.3127, p = 0.1198, respectively). This may have been because 26 eyes was too small a sample. There was no significant difference of the VEGF level between the 26 eyes that received retinal photocoagulation and the 28 eyes without retinal photocoagulation (p = 0.3634).

Analysis of the 25 patients who did not have cataract surgery revealed that the vitreous level of vascular endothelial growth factor was not associated with improvement of macular edema or improvement of visual acuity (r = 0.2904, p = 0.1568 and r = - 0.1856, p = 0.3633, respectively), also possibly because of the small sample size. There were no significant differences of VEGF levels, improvement of macular edema, or improvement of visual acuity when we compared the group that did not undergo cataract surgery with the group that had cataract surgery (p = 0.5093, p = 0.8731, and p = 0.4946, respectively).

## Discussion

The present study demonstrated that the vitreous level of VEGF was significantly higher in BRVO patients who showed less improvement of their best-corrected visual acuity after PPV (Figure 1). As a result, the VEGF level had a significant influence on the visual outcome according to multivariate analysis. These findings suggest that measurement of VEGF in the vitreous fluid may be useful for predicting the visual prognosis after PPV. In contrast to our findings, Shimura et al.[12] have reported that the visual prognosis is not correlated with the vitreous level of VEGF. However, they did not perform retinal photocoagulation, while this was done in the present study. Retinal photocoagulation has been shown to induce the expression of various cytokines, such as interleukin (IL)-1. β, IL-6, IL-8, and VEGF, [13,14] suggesting that the effect of photocoagulation on the production of VEGF may explain the difference between their findings and ours.

The Branch Vein Occlusion Study was a multicenter randomized clinical trial that established guidelines for use of retinal photocoagulation in the treatment of macular edema. The effectiveness of argon laser photocoagulation was demonstrated, but it was not recommended within 3 months of the onset of BRVO because spontaneous improvement can occur[15]. However, some patients have poor visual acuity and persistent macular edema despite photocoagulation. Recently, PPV combined with posterior vitreous detachment has been reported to effectively reduce macular edema and improve visual acuity in BRVO patients[16,17]. Therefore, we performed vitrectomy more than 3 months after the onset of BRVO in patients who had clinically detectable diffuse or cystoid macular edema, or who had persistent macular edema after photocoagulation. We also performed cataract surgery in 29 patients, because our subjects were relatively old with an average age of 63.1 years.

The macula is important for detailed vision, especially the fovea that consists entirely of cones[18]. In humans, histological studies have shown that macular edema is associated with swelling of the Müller cells, especially in the outer plexiform layer of the neurosensory retina [19-21]. Accordingly, macular edema may affect retinal function and lead to visual impairment in BRVO patients.

The present study demonstrated that a higher VEGF level in the vitreous fluid at the time of surgery was significantly associated with more marked improvement of macular edema after PPV, which was in agreement with our findings in a previous study of PPV for BRVO[7]. The vitreous level of VEGF was also significantly correlated with the improvement of BCVA and the decrease of foveal thickness. Intravitreal injection of VEGF has been reported to cause retinal edema, dilated and tortuous vessels, and capillary closure in adult primates, [22] while treatment with bevacizumab (a monoclonal antibody targeting VEGF) or ranibizumab (an Fab fragment that binds and neutralizes all isoforms of VEGF-A) improves macular edema in patients with BRVO [23-25]. Therefore, reduction of the intraocular VEGF level may be one of the mechanisms by which PPV improves macular edema in BRVO patients. In agreement with this hypothesis, we previously found that the vitreous level of VEGF was lower at the time of repeat vitrectomy in patients with macular edema due to retinal vein occlusion[26].

Interestingly, the improvement of visual acuity and the improvement of macular edema did not occur in parallel. Although the retinal thickness at the central fovea has been reported to influence visual acuity, [27,28] we found that acuity sometimes showed little improvement even if macular edema resolved. This may have been because 1) improvement of visual acuity required longer than the 6-month follow-up period of this study or 2) improvement of vision failed to occur after edema resolved because of permanent photoreceptor cell damage due to macular ischemia in patients with high VEGF levels. Foveal bleeding could also influence the visual prognosis. Accordingly, a larger prospective and randomized study is needed to clarify the relation between visual acuity and macular edema in patients with BRVO.

In the present study, multivariate analysis showed that the vitreous VEGF level and retinal photocoagulation were significantly correlated with improvement of visual acuity and with the decrease of foveal thickness. Aiello et al.[9] previously reported that the vitreous level of VEGF was reduced by retinal photocoagulation, and our findings would suggest that the decrease of VEGF due to retinal photocoagulation results in improvement of BCVA and a decrease of foveal thickness. However, the relations between retinal photocoagulation, the vitreous level of VEGF, improvement of visual acuity, and improvement of macular edema in patients with BRVO need to be investigated further.

The present study had several limitations. One major problem was the performance of laser photocoagulation prior to surgery. For the following reasons, we performed a combined analysis of the groups with (n = 26) and without (n = 28) laser photocoagulation. The first reason is that there was no significant difference of the VEGF level between the 26 eyes that received retinal photocoagulation and the 28 eyes without it (data not shown, p = 0.6755). Secondly, laser photocoagulation increases the expression of various cytokines, including VEGF, by cultured human retinal pigment epithelial (RPE) cells, but upregulation of VEGF occurs from 6 hours after photocoagulation and there is a return to the basal level by 72 hours[29]. In contrast, Itaya et al.[30] found the maximum VEGF level on day 3 *in vivo*, coinciding with the peak of macrophage infiltration. They suggested that the difference from *in vitro *data arose because infiltrating macrophages contributed more to upregulation of VEGF than RPE cells or because secretion of VEGF was induced by the interaction of macrophages with RPE cells. Furthermore, changes of retinal VEGF mRNA expression after laser photocoagulation were confined to RPE cells in miniature pigs, with reduced mRNA expression immediately after photocoagulation and a return to normal by 42 days[31]. These results suggest that any increase of VEGF expression in the retina after photocoagulation is transient, and that the VEGF level decreases again relatively fast and then becomes stable. Because the average interval was 2.3 ± 1.5 months from laser photocoagulation to vitrectomy, photocoagulation is unlikely to have influenced VEGF levels. Another limitation of this study was the performance of cataract extraction at the time of vitrectomy. However, there were no significant differences of VEGF levels, improvement of macular edema, or improvement of visual acuity between the patients undergoing cataract surgery and those who did not. Multiple regression analysis demonstrated that cataract surgery was not correlated with the improvement of macular edema or the improvement of visual acuity. Therefore, cataract surgery did not influence our results. Further investigations will be needed to clarify the relation among the vitreous level of VEGF, improvement of macular edema, and improvement of visual acuity, as well as the possible effects of laser photocoagulation and cataract surgery.

## Conclusions

The present study showed that the vitreous level of VEGF was significantly higher in BRVO patients with less improvement of best-corrected visual acuity after PPV. In contrast, patients with high vitreous VEGF levels displayed greater improvement of macular edema than those with low VEGF levels. These results suggest that VEGF in the vitreous fluid might influence both the visual prognosis and the response of macular edema to vitrectomy in patients with BRVO.

## Financial support

None.

## Conflict of interests

The authors declare that they have no competing interests.

## Authors' contributions

HN, and HF were involved in the design and conduct of the study. Collection and management of the data were done by HN, and SE, while analysis and interpretation of the data were performed by HN, HF, TM, and KS. Preparation of the first draft of the manuscript was done by HN, and review and approval of the manuscript was performed by HF, TM, and SE.

## Pre-publication history

The pre-publication history for this paper can be accessed here:

http://www.biomedcentral.com/1471-2415/10/11/prepub
